# The Attitudes of Healthcare Workers Towards Cardiopulmonary Resuscitation Training in Qassim, Saudi Arabia

**DOI:** 10.7759/cureus.92418

**Published:** 2025-09-16

**Authors:** Abdullah Altulayhi, Yasir A Alrusayni, Mohammed Alhnaya

**Affiliations:** 1 Emergency Medicine Department, King Fahd Specialist Hospital, Buraydah, SAU

**Keywords:** cpr, emergency response, healthcare professionals, life-saving, perceptions, preparedness, resuscitation

## Abstract

Background

Cardiopulmonary resuscitation (CPR) is a critical life-saving skill for healthcare workers. Understanding their attitudes towards CPR training is essential for improving emergency response capabilities.

Objective

To assess the attitudes, perspectives, and preparedness of healthcare professionals regarding CPR training and its practical implementation in Qassim, Saudi Arabia.

Methods

A cross-sectional study was conducted using an online structured questionnaire. The study targeted a sample size of 400 healthcare workers. The questionnaire was adopted using a forward-backward translation protocol and pretested for validity. Data were analyzed using IBM SPSS Statistics for Windows, Version 27 (Released 2020; IBM Corp., Armonk, New York, United States), employing descriptive statistics, Mann-Whitney, and Kruskal-Wallis tests for statistical differences.

Results

Among 172 healthcare workers surveyed in Qassim, Saudi Arabia, 95.9% had prior CPR training. While participants generally reported confidence in basic CPR tasks, 55.2% expressed fears when performing CPR, primarily concerned by unsuccessful outcomes (39.0%) and causing rib fractures (23.3%). Despite these apprehensions, 89.0% indicated they would not delay or avoid performing CPR. Participants demonstrated a strong commitment to CPR, with high willingness to perform it on family members (81.4%), colleagues (69.8%), and the public (66.9%). They strongly endorsed increasing public awareness activities (73.8%) and making CPR courses freely accessible (69.2%). The median perceived difficulty level for learning CPR was three on a 10-point scale.

Conclusion

Healthcare workers in Qassim demonstrated a strong commitment to CPR, despite some apprehensions. Recommendations for improving CPR training and public accessibility were identified, which could inform policy development and enhance emergency care outcomes in the region.

## Introduction

Medical crises involving the heart and lungs need a rapid and efficient response [1]. In the event of a life-threatening emergency, such as a cardiac arrest, cardiopulmonary resuscitation (CPR) is an essential life-saving procedure that keeps blood flowing to the brain and other critical organs [2]. Heart disease and lung disease are major causes of death and disability on a global scale. Responding to unexpected cardiac events and respiratory failures requires healthcare providers to possess basic competence in CPR, which is recognized globally [3].

Responding to medical crises is a critical responsibility of healthcare personnel [4]. How healthcare providers feel about CPR training has a direct bearing on how well they are able to save lives during emergencies, which in turn affects the results for patients [5]. The study's backdrop highlights the worldwide significance of CPR in emergency response, the influence of healthcare personnel on patient outcomes, and the need for context-specific knowledge to enhance training programs [6]. Views on CPR differ among healthcare professionals and non-healthcare individuals across various settings and countries. Regular CPR training is crucial for maintaining competence and reducing anxiety among healthcare professionals [7]. To enhance overall CPR knowledge and willingness to perform the procedure, it is essential to boost CPR training and awareness within the general population [8].

Investigating the attitudes of healthcare personnel towards CPR training is essential for enhancing emergency response capabilities and contributing valuable insights to global health research. This study aims to assess and understand the attitudes, perspectives, and preparedness of healthcare professionals about CPR training and its practical implementation in Qassim, Saudi Arabia. The findings of this research have the potential to inform the development of more effective policies and initiatives, ultimately benefiting both healthcare providers and the communities they serve. By identifying potential barriers, perceptions, and factors influencing CPR performance, this study can contribute to improving training programs and emergency care outcomes in the region.

## Materials and methods

Study design

This was a cross-sectional study that utilized an online structured questionnaire to assess the attitudes of healthcare workers toward CPR training in the Qassim region.

Study settings

The study focused on healthcare workers who had undergone CPR training in healthcare facilities in the Qassim region, including hospitals, clinics, and medical centers. Participants were invited to complete the online questionnaire (Google Forms, Google LLC, California, US) through various communication channels, such as email, WhatsApp, and professional social media platforms. The data collection period was from January 2024 to April 2024.

Study population

The target population for this study was healthcare workers in the Qassim region. The online survey was designed and distributed to eligible participants, including physicians, nurses, paramedics, and other healthcare professionals. All healthcare workers who had received CPR training in the month before data collection began (i.e., Dec 2023) and were 18 years of age or older, regardless of gender, were eligible to participate. Individuals who were unable to understand and respond to the questionnaire in Arabic or those who declined to participate were excluded from the study.

To determine the sample size, we used a cross-sectional study calculator (Raosoft Inc., Seattle, WA, US) with a 5% error margin, a 95% confidence interval, and a conservative estimated distribution rate. Based on the estimated population of healthcare workers in the Qassim region, a sample size of 400 participants was targeted. To ensure a representative sample and increase the statistical power of the study, the sample size was increased as necessary.

Convenient sampling technique was employed to recruit healthcare workers who had undergone CPR training in the Qassim region. The online questionnaire included a statement and specific items to confirm the eligibility of the participants.

Data collection

Data was collected using an online, structured questionnaire administered through a secure and reliable platform. Participants were provided with clear instructions and explanations regarding the study's objectives and the confidentiality of their responses. The questionnaire was available in Arabic and included questions related to participants' demographics, attitudes towards CPR training, and their sources of information.

A recently pretested questionnaire was utilized for data collection [9]. The questionnaire included inquiries about the participants' demographics, and modifications were made to enhance the validity of the adopted version. To ensure accuracy, the translated Arabic version of the questionnaire was developed using a forward-backward translation protocol, with the aid of bilingual Arabic and English speakers who were unfamiliar with the original questionnaire. Additionally, questions were included to assess specific aspects of public awareness and inquire about the sources of information.

## Results

A total of 172 healthcare workers participated in this study. The majority were male participants (66.9%), single (70.3%), and held a bachelor's degree (79.7%). The median age of the participants was 27 years (IQR: 25-30), with a range from 20 to 64 years. A high percentage (95.9%) of participants had undergone a formal CPR training course before the study.

The detailed demographics of the participants are shown in Table 1.

**Table 1:** Demographic characteristics and CPR training status of the study participants (n=172) CPR: Cardiopulmonary resuscitation

Variable	Groups	N	%
Gender	Male	115	66.90%
	Female	57	33.10%
Marital status	Single	121	70.30%
	Married	51	29.70%
Educational level	Diploma	3	1.70%
	Bachelor	137	79.70%
	Higher education	32	18.60%
Income (Saudi Riyals)	<5,000	16	9.30%
	5,000-10,000	50	29.10%
	10,001-15,000	34	19.80%
	>15,000	72	41.90%
Have you undergone CPR training before today?	Yes	165	95.90%
	No	7	4.10%

Regarding the perceived difficulty of CPR tasks, the majority of participants reported no difficulty in recognizing no response (74.4%) and no breathing (59.3%), as well as locating the hand position (59.3%). However, chest compressions and mouth-to-mouth breathing were perceived as more challenging, with 47.1% and 42.4% reporting no difficulty, respectively. Notably, 12.2% of participants found chest compressions to be the most difficult task, while 15.7% considered mouth-to-mouth breathing the most challenging. The levels of difficulties in each CPR practice are detailed in Table 2.

**Table 2:** Perceived difficulty of CPR tasks among healthcare workers in Qassim, Saudi Arabia CPR: Cardiopulmonary resuscitation

Task	No difficulty	4th most difficult	3rd most difficult	2nd most difficult	Most difficult
Recognizing no response	128 (74.4%)	16 (9.3%)	17 (9.9%)	4 (2.3%)	7 (4.1%)
Recognizing no breathing	102 (59.3%)	36 (20.9%)	17 (9.9%)	13 (7.6%)	4 (2.3%)
Locating hand position	102 (59.3%)	31 (18.0%)	29 (16.9%)	7 (4.1%)	3 (1.7%)
Chest compressions	81 (47.1%)	30 (17.4%)	17 (9.9%)	23 (13.4%)	21 (12.2%)
Mouth-to-mouth breathing	73 (42.4%)	26 (15.1%)	25 (14.5%)	21 (12.2%)	27 (15.7%)

The study revealed that a high proportion of healthcare workers (55.2%) had fears when performing CPR. The most common concerns were fear of unsuccessful outcomes (39.0%) and causing rib fractures (23.3%). Other fears included low level of confidence (15.1%), fear of acquiring infections (16.9%), and legal repercussions of an adverse outcome (15.1%). Despite these fears, the vast majority of participants (89.0%) indicated that they would not delay or avoid performing CPR due to these concerns. This suggests a strong commitment to providing life-saving interventions despite personal apprehensions (Table 3).

**Table 3:** Prevalence of fears and concerns among healthcare workers regarding CPR performance CPR: Cardiopulmonary resuscitation

Statement	No		Yes	
	N	%	N	%
Do you have any fears when doing CPR?	77	44.8%	95	55.2%
Low level of confidence	146	84.9%	26	15.1%
Fear of unsuccessful outcome	105	61.0%	67	39.0%
Fear of causing rib fractures	132	76.7%	40	23.3%
Fear of acquiring infections	143	83.1%	29	16.9%
Aversion to doing mouth-to-mouth breathing	154	89.5%	18	10.5%
Uncomfortable with doing CPR	164	95.3%	8	4.7%
Legal repercussions of an adverse outcome	146	84.9%	26	15.1%
Does not want to resuscitate opposite gender	167	97.1%	5	2.9%

Participants provided valuable recommendations for improving CPR training and public accessibility in Qassim, Saudi Arabia. The most frequent suggestions were increasing the number and frequency of public awareness activities (73.8%), making CPR courses free to the public (69.2%), and promoting CPR for all through various public and private institutions (66.9%). Additionally, 62.8% of participants recommended implementing CPR training in all schools. These findings indicate a strong desire among healthcare workers to expand CPR knowledge and skills throughout the community (Table 4).

**Table 4:** Participant-reported recommendations for improving CPR training and public accessibility in Qassim, Saudi Arabia CPR: Cardiopulmonary resuscitation; AED: Automated external defibrillator.

Statement	No		Yes	
	N	%	N	%
Increase number and frequency of public awareness activities	45	26.2%	127	73.8%
Promote CPR for all through public and private institutions	57	33.1%	115	66.9%
Make CPR courses free to the public	53	30.8%	119	69.2%
Implement CPR training in all schools	64	37.2%	108	62.8%
Make CPR courses easily accessible	74	43.0%	98	57.0%
Have Good Samaritan Laws to help the rescuer	105	61.0%	67	39.0%
Make training equipment easily available to the public	74	43.0%	98	57.0%
Make AEDs available in more areas in the country	92	53.5%	80	46.5%
Provide face-shields to all participants of CPR courses	121	70.3%	51	29.7%

When asked about their willingness to perform CPR on different categories of cardiac arrest victims, the majority of participants reported they would perform both chest compressions and mouth-to-mouth ventilations at a 30:2 ratio until the ambulance arrives. This was highest for family members (81.4%), followed by work colleagues (69.8%) and members of the public (66.9%). Interestingly, a quarter of participants would perform only chest compressions for work colleagues (25.6%) and members of the public (26.2%). The results are shown in Table 5.

**Table 5:** CPR interventions by healthcare workers for different categories of cardiac arrest victims in Qassim, Saudi Arabia CPR: Cardiopulmonary resuscitation

If you encounter someone undergoing a cardiac arrest, which of the following would you perform?	Family member	Work colleague	Public
Only chest compressions till the ambulance arrives	22 (12.8%)	44 (25.6%)	45 (26.2%)
Only mouth-to-mouth ventilation till the ambulance arrives	6 (3.5%)	6 (3.5%)	5 (2.9%)
Both chest compressions and mouth-to-mouth ventilation at 30:2 till the ambulance arrives	140 (81.4%)	120 (69.8%)	115 (66.9%)
None of the above	4 (2.3%)	2 (1.2%)	7 (4.1%)

The study also explored motivations and barriers for CPR refresher training. A high percentage of participants agreed to undergo CPR refresher training with recertification in two years. The primary motivations were to refresh and update life-saving skills and continue to help others and save lives in an emergency (68.6%). Recertification requirements for job purposes were also an important factor (64.5%). Only a small proportion of participants cited barriers such as feeling that learning the skill once was enough (4.1%) or enjoyed attending the training with friends (7.0%). The results are shown in Table 6.

**Table 6:** Motivations and barriers for CPR refresher training among healthcare workers in Qassim, Saudi Arabia CPR: Cardiopulmonary resuscitation

Why would you agree to go for CPR refresher training with recertification in two years?	No		Yes	
	N	%	N	%
Yes, I agree				
To refresh my memory and update my skills to help save a life in an emergency	54	31.4%	118	68.6%
I need the CPR certification as part of my compulsory core-curricular activity	61	35.5%	111	64.5%
I need to renew my CPR certification	90	52.3%	82	47.7%
No, I do not agree				
I feel that having already learning the skill once is enough	150	87.2%	22	12.8%
I feel a refresher course would be too troublesome to organize and attend	165	95.9%	7	4.1%
I feel I would not have the time to attend a refresher	161	93.6%	11	6.4%

Our study participants showed a strong inclination to recommend CPR training to family members and the public. An overwhelming majority stated they would recommend that other family members learn CPR. All participants agreed that members of the public should learn CPR. These findings are further detailed in Table 7.

**Table 7:** Recommendations from healthcare workers for CPR training among family members and the public in Qassim, Saudi Arabia CPR: Cardiopulmonary resuscitation

Statement	No		Yes	
	N	%	N	%
Would you recommend that other members of your family learn CPR?	2	1.2%	170	98.8%
Who would you recommend to learn CPR? (Brothers)	8	4.7%	164	95.3%
Who would you recommend to learn CPR? (Sisters)	31	18%	141	82.0%
Who would you recommend to learn CPR? (Parent)	59	34.3%	113	65.7%
Would you recommend that other members of the public learn CPR?	0	0%	172	100.0%
Would you recommend that other members of the public learn CPR? (Colleague)	99	57.6%	73	42.4%
Would you recommend that other members of the public learn CPR? (Boss)	102	59.3%	70	40.7%
Would you recommend that other members of the public learn CPR? (All public)	6	3.5%	166	96.5%

Finally, the study examined the perceived difficulty in learning CPR among the different demographic groups. The median difficulty level reported was 3 (IQR: 1-5) on a scale of 1-10 (where 1 is not difficult at all and 10 is very difficult). There were no statistically significant differences in perceived difficulty based on gender or marital status. However, there was a trend towards higher perceived difficulty among those with only a high school education compared to those with higher education levels, although this difference did not reach statistical significance. These results suggest that CPR training is generally perceived as moderately challenging across different demographic groups, with potential for tailored approaches for those with lower educational levels (Table 8).

**Table 8:** Comparison of perceived difficulty in learning CPR among different demographic groups of healthcare workers in Qassim, Saudi Arabia CPR: Cardiopulmonary resuscitation; IQR: Interquartile range; Difficulty scale: 1 (Not difficult at all) to 10 (Very difficult)

Variable	Group	The level of difficulty in learning CPR was	
		Median (IQR)	P-value
Gender	Male	3 (1-5)	0.102
	Female	4 (2-5)	
Marital status	Single	3 (1-5)	0.789
	Married	2 (2-5)	
Educational level	Diploma	5 (2-6)	0.266
	Bachelor	3 (2-5)	
	Higher education	2 (1-4)	
Income (Saudi Riyals)	<5,000	3.5 (1.5-4.5)	0.934
	5,000-10,000	3 (1-5)	
	10,001-15,000	3 (1-5)	
	>15,000	3 (2-5)	
Have you undergone CPR training before today?	Yes	3 (1-5)	0.284
	No	4 (3-5)	

## Discussion

Our study utilized an online structured questionnaire to evaluate and comprehend the attitudes and level of awareness regarding CPR among healthcare professionals in Qassim. According to the study, most participants considered basic CPR techniques, such as identifying response and breathing, to be quite simple, whereas performing mouth-to-mouth breathing and chest compressions were more challenging. Despite concerns about poor outcomes, rib fractures, infections, and legal consequences, the vast majority of participants were determined to conduct CPR. They actively advocated for enhancing CPR awareness and training for the public, suggesting efforts such as free courses and incorporating CPR instruction in schools. Participants were willing to conduct chest compressions and ventilations, especially for family members; however, some preferred chest compressions for non-family members. Motivations for refresher training were predominantly driven by the need to update life-saving skills, with few indications of impediments, including a feeling that learning the skill once was enough or enjoying attending the training with friends. Overall, CPR was somewhat difficult, especially for individuals with lower education levels, but the healthcare workers demonstrated a strong dedication to enhancing CPR knowledge and practice.

Our study revealed that the most feared factor while performing CPR was unsuccessful outcomes. A survey conducted among in-hospital healthcare professionals found that in the event of a cardiac arrest, the majority of participants were confident in their CPR expertise (86%), knew what to do, and could take command if necessary [7]. In the most recent real-life CPR situation, 30% were concerned about making mistakes or causing difficulties, while 57% were anxious, and 27% were apprehensive [7]. These results align with our data. Despite being a less feared problem in the present study, investigations conducted globally indicate that infection transmission is still a concern. A cross-sectional study of healthcare workers and trainees in the United States and Saudi Arabia, using an internet survey, showed that concern about developing COVID-19 was the key demotivating factor for 74% of the population [2]. In another study done at Swedish hospitals, healthcare professionals were asked to answer a questionnaire before and after CPR training. Results showed that practitioners who conducted CPR in real life reduced their fear of infection transmission from 90% to 14%. While among nurses, fear was reduced from 82% before the training to 17% after it [10].

In a study conducted on public attitudes towards CPR training and performance in Singapore, results showed that the most challenging skills were mouth-to-mouth breathing and chest compressions, whereas the easiest was recognizing non-responsiveness [9]. These results support our findings, which show that there is no difficulty in identifying response (74.4%), while 12.2% and 15.7% of the study population found chest compressions and mouth-to-mouth breathing, respectively, most challenging. Families were the group most willing to perform CPR (81.4%), followed by work colleagues (69.8%) and members of the public (66.9%) in the present survey. However, a cross-sectional study found that people were hesitant to do mouth-to-mouth resuscitation in an emergency, but will perform chest compression with the greatest rate being (66%) on a stranger, followed by (58%) on a friend or coworker, and (36%) on a family member (MSc thesis: Zaayman H. A description of the knowledge and attitudes towards bystander CPR amongst participants in a community outreach initiative in Cape Town. Stellenbosch University; 2021). There was no significant difference based on gender or marital status regarding how hard people found CPR. However, participants with a high school education tended to find CPR more difficult than those with higher education, although this difference was not statistically significant. The research from Singapore also reported that gender and marital status had no significant influence on perceived difficulty in CPR instruction, differences in education levels indicate a possible need for specialized training techniques [9]. These could include additional hands-on practice and support for people with lower educational backgrounds to develop their confidence and competence [9].

Refreshing and updating life-saving skills were found to be the most motivating factor (68.6%) for refresher training in CPR. These findings are consistent with findings from another study, which found that the majority of participants (69.7%) agreed to attend refresher training every two years and 88.7% thought that everyone should be trained in CPR [9]. Although stated to be modest, barriers like the belief that mastering a skill only once is sufficient (4.1%) are acknowledged as a global issue. According to a survey among several paramedics, all doctors believed that CPR was essential, but only anesthesiologists knew how often CPR guidelines were updated. Roughly half of physicians, (48%) of surgeons and (77%) of anesthesiologists stated that they had updated their CPR training in the previous five years [11]. A qualitative analysis of a national multicenter randomized clinical trial revealed that having family members present during resuscitation gives emotional closure and psychological healing for those affected [12]. Similarly, participants from the present research agreed that family members and the public should learn CPR. This viewpoint is consistent with the prevailing opinion that family members and the public should actively participate in CPR instruction. Furthermore, family members observing CPR has been connected with decreased post-traumatic stress in some circumstances [12]. Nearly 73.8% of our study participants recommended increasing the quantity and frequency of public awareness campaigns. A similar response was noted in a study conducted among final-year university students in Nigeria. A majority of the respondents thought that CPR education ought to be included in the school curriculum [13].

To enhance overall CPR knowledge and encourage willingness to perform the procedure, it might be essential to boost CPR training and awareness within the general population [8]. Overall, medical students exhibited good knowledge and positive attitudes but poor practice [14]. While CPR is generally perceived as moderately challenging across demographic groups, simplifying content or providing additional support may enhance learning and skill retention for those with lower educational backgrounds. The strong support for increasing public awareness activities and making CPR courses free to the public highlights the need for widespread public education on CPR. Training should place greater emphasis on the hands-on components of CPR, with more time spent on mastering chest compressions and mouth-to-mouth techniques to ensure healthcare workers are fully prepared for real-life situations [15]. Promoting CPR training to non-healthcare populations can create a more resilient community, with more bystanders equipped to provide life-saving interventions [16]. By expanding access to CPR training, particularly in schools and public institutions, there is a potential to improve the general population’s ability to respond in situations of emergencies, contributing to better cardiac arrest survival rates in the community [17].

## Conclusions

This study aimed to assess the attitudes, perspectives, and preparedness of healthcare workers in Qassim, Saudi Arabia, regarding CPR training and its practical implementation. Most healthcare workers feel confident in recognizing the need for CPR and performing basic tasks like locating hand positions. Challenges remain in executing more complex procedures such as chest compressions and mouth-to-mouth breathing. Despite fears of unsuccessful outcomes and rib fractures, the vast majority of participants expressed a strong commitment to performing CPR in emergencies. Suggestions such as increasing public awareness, offering free CPR courses, and integrating CPR training in schools demonstrated a strong desire to expand life-saving knowledge within the community. Our results also suggest that while CPR training is generally well-received, tailored approaches may be needed for those with lower educational backgrounds to further improve confidence and skills. Future studies should focus on longitudinal designs and diverse sampling methods to explore how these attitudes and skills evolve over time and across different populations.
